# Clinical benefit of neoadjuvant anti‐PD‐1/PD‐L1 utilization among different tumors

**DOI:** 10.1002/mco2.61

**Published:** 2021-03-11

**Authors:** Zhiyang Li, Xin Wu, Yanjie Zhao, Yinan Xiao, Yunuo Zhao, Ting Zhang, Hui Li, Fushen Sha, Yating Wang, Lei Deng, Xuelei Ma

**Affiliations:** ^1^ Department of Biotherapy State Key Laboratory of Biotherapy, West China Hospital Sichuan University Chengdu Sichuan China; ^2^ West China Hospital, West China School of Medicine Sichuan University Chengdu Sichuan China; ^3^ State Key Laboratory of Biotherapy Department of Biotherapy, West China Hospital, Cancer Center Sichuan University Chengdu Sichuan China; ^4^ Head and Neck Carcinoma Department, Radiation Oncology Department, Cancer Center West China Hospital Chengdu Sichuan China; ^5^ Jacobi Medical Center Albert Einstein College of Medicine, Bronx New York New York USA; ^6^ Department of Internal Medicine State University of New York: Downstate Medical Center Brooklyn New York USA; ^7^ Department of Internal Medicine Louis A. Weiss Memorial Hospital Chicago Illinois USA

**Keywords:** atezolizumab, lung cancer, neoadjuavant therapy, PD‐1/PD‐L1 inhibitors, triple‐negative breast cancer

## Abstract

PD‐1/PD‐L1 (programmed cell death‐1 and programmed death‐ligand 1) inhibitors utilization in neoadjuvant therapy has been assessed in tumors. This study focused on the clinical benefits of neoadjuvant anti‐PD‐1/PD‐L1 therapy. A comprehensive search was conducted in electronic databases to identify eligible studies. Major response rate (MRR) and complete response rate (CRR) were pooled in this analysis to assess the efficacy of neoadjuvant anti‐PD‐1/PD‐L1 utilization, all grades and high‐grade adverse events (AEs) were pooled to evaluate its safety. Twenty studies were included in this meta‐analysis, with 828 patients suffering from different tumors. The pooled CRR of triple‐negative breast cancer was 0.569 (95% CI 0.514, 0.624, *I* ^2^ = 0%) and the pooled MRR of lung cancer was 0.471 (95% CI 0.267, 0.575, *I* ^2^ = 0%). The most frequent adverse event was fatigue (0.272 95% CI 0.171, 0.402, *I* ^2^ = 87%), and the most common high‐grade adverse event was febrile neutropenia (0.084 95% CI 0.063, 0.112, *I* ^2^ = 85%). In conclusion, neoadjuvant anti‐PD‐1/PD‐L1 therapy received satisfactory clinical results in these tumors included.

## Graphical abstract

Twenty studies were included in this meta‐analysis. The pooled CRR of triple‐negative breast cancer and urothelial carcinoma was 0.569 (95% CI 0.514, 0.624) and 0.320 (95% CI 0.234, 0.407), respectively. The pooled MRR of lung cancer was 0.471 (95% CI 0.367, 0.575). In conclusion, neoadjuvant anti‐PD‐1/PD‐L1 therapy received satisfactory clinical results in these tumors.

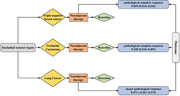

## 1 INTRODUCTION

Cancer poses threats to the health of human beings worldwide. Global cancer statistics in 2018 revealed that 18 million people suffered from all sorts of cancer and reported 9 million fatalities.^1^ Some advanced cancer patients were not suitable for surgery, the optimal treatment for removing tumors. In recent years, neoadjuvant therapy has shown its efficiency of downstaging and local control.^2^, ^3^, ^4^, ^5^, ^6^, ^7^ Therefore, this therapy provided an opportunity for advanced cancer patients to extend the indication of surgery. However, a primary concern of this therapy was the risk of failure. Tumors, such as local advanced lung cancer can be removed by surgery. The tumor downstaging brought better survival outcome and lower recurrence rate. However, patients who failed to respond after neoadjuvant therapy will suffer cancer progression and thus miss the last chance of receiving radical treatment. As a result, it is fundamental to pursue efficient drugs to decrease the possibility of neoadjuvant therapy failure.

Immune checkpoint inhibitors (ICIs) have been established as effective drugs for several cancers.^8^, ^9^, ^10^, ^11^ Recent years have witnessed a blowout of ICIs, containing cytotoxic T‐lymphocyte‐associated protein 4 (CTLA‐4) and programmed cell death protein 1/programmed death‐ligand 1 (PD‐1/PD‐L1) inhibitors. Programmed cell death‐1 protein (PD‐1), a significant immune checkpoint, can repress the T‐cell immune response. Some tumor cells can secrete programmed death‐ligand 1 (PD‐L1) to combine with PD‐1 molecule on the T cells. This process can suppress the activity of T cells and enhance the immune ignorance of tumor cells.^12^, ^13^ By preventing the combination of PD‐1 and PD‐L1, PD‐1/PD‐L1 inhibitors can reduce the immune escape of tumor cells. Consequently, they can enhance the antitumor function of immune system. PD‐1/PD‐L1 inhibitors like atezolizumab and pembrolizumab showed superior efficacy in clinical utilization.^12^, ^14^, ^15^, ^16^, ^17^, ^18^, ^19^ Some studies try to uncover the clinical value of the utilization of PD‐1/PD‐L1 inhibitors in neoadjuvant therapy.^20^, ^21^, ^22^, ^23^, ^24^, ^25^, ^26^, ^27^, ^28^, ^29^, ^30^, ^31^, ^32^, ^33^, ^34^, ^35^, ^36^, ^37^, ^38^, ^39^ To evaluate the efficacy and safety of the therapy, this meta‐analysis pooled and analyzed the data of these trials.

## 2 METHODS

### 2.1 Search strategy

This systemic review and meta‐analysis was conducted following the Preferred Reporting Items for Systematic Reviews and Meta‐Analysis (PRISMA) guideline, and all the basic characteristics were included.^40^

A systematic search of both single‐arm and double‐arm trials of neoadjuvant anti‐PD‐1/PD‐L1 therapy was performed in PubMed from inception to November 2020. The keywords “neoadjuvant,” “PD‐1,” and “PD‐L1” were used in the searches, and the Medical Subject Headings (MeSH) terms of the above search keywords were combined in the search strategy: ((((PD AND 1) OR CD297) AND (Receptor OR Antigen]) OR (Programmed Cell Death 1 Protein) OR PD1) OR ((((B7 AND H1) OR CD274) AND Antigen) OR (((B7 AND H1) OR (PD AND L1)) AND (Costimulatory Protein OR Immune Costimulatory Protein)) OR (Programmed Cell Death 1 Ligand 1 Protein)) OR PD‐L1)) AND ((Neoadjuvant AND ((Therapy OR Therapies) OR Treatment)) OR Neoadjuvant). To detect any missive study, the references of the included articles and the published meta‐analysis and systematic review were assessed manually. The initial search for the potentially eligible studies was performed by screening the title and the abstract independently by two authors (Zhiyang Li, Lei Deng), and any divergence was resolved by a third reviewer (Xuelei Ma).

### 2.2 Selection criteria

Eligible studies must satisfy the predefined criteria: (1) study investigated the efficacy or the safety of the application of PD‐1/PD‐L1 inhibitors in neoadjuvant therapy; (2) study included no less than 10 patients; (3) study reported the outcome of pathologic complete response (pCR) or the adverse events (AEs); (4) study was published in English. Studies would be excluded if (1) full‐length article of study cannot be found; (2) ongoing study did not report current data; (3) study reported data which were overlapped by larger sample size article (studies with same register number). The full texts were evaluated by two independent authors (Fushen Sha, Yanjie Zhao) according to the inclusion and exclusion criteria, and any discrepancy was resolved by discussion, and binding verdict in case of disagreement in the discussion was given by a third author (Xuelei Ma).

### 2.3 Data extraction and quality assessment

Two authors (Xin Wu, Zhiyang Li) extracted data from eligible articles to predefined extraction forms independently. Any disagreement in data‐extracting process was resolved by a third senior reviewer (Xuelei Ma). The primary endpoints were the pathologic response outcomes (pathologic completed response and major pathologic response) and the second endpoint was any‐grade AEs and high‐grade AEs (grades 3–5) during neoadjuvant treatment. The pathologic response was assessed by the Response Evaluation Criteria in Solid Tumors (RECIST v1.1), and the AEs were assessed according to the Common Terminology Criteria for Adverse Events (CTCAE).^41^, ^42^ Additionally, the following baseline details in all the eligible articles were extracted if available: author, publication year, study design, sample size, registration number, age, cancer type, the regimen of therapy, and PD‐L1 positive rate. The PD‐L1 positive was defined as the percentage of PD‐L1‐positive cells exceeding 1%.^16^

Cochrane Collaboration guidelines were used to evaluate the methodological quality of the included randomized controlled trial (RCT).^43^ Six domains were assessed in this tool (selection bias, performance bias, detection bias, attrition bias, reporting bias, and other bias). Each domain could be assessed as low risk, high risk, or unclear risk. And the quality of nonrandomized trials was evaluated according to the Methodological Index for Nonrandomized Studies (MINORS) score.^44^ This MINORS tool contained eight items for the nonrandomized studies. Each item was scored as 0 (not reported), 1 (reported but inadequate), and 2 (reported and adequate). In this meta‐analysis, studies with scores <9 were considered as low quality. Methodological quality of the included trials was assessed independently by two authors (Ting Zhang, Yinan Xiao).

### 2.4 Statistical analysis

Review Manager 5.3 and OpenMeta‐Analyst were used to analyze data.^45^, ^46^ This meta‐analysis is a single‐arm analysis, so event rate and 95% confidence interval (CI) were calculated as evaluation indexes for the efficacy and safety of neoadjuvant anti‐PD‐1/PD‐L1 therapy for complete response rate (CRR), major response rate (MRR), any‐grade AEs, and high‐grade AEs. Cochran's *Q* test and *I* ^2^ test were performed to assess the heterogeneity among studies, *p *< .05 or *I* ^2^ > 50% indicated the existence of obvious heterogeneity, then random effect model was selected when pooling data.^47^

## 3 RESULT

### 3.1 Study selection and characteristics

**FIGURE 1:**
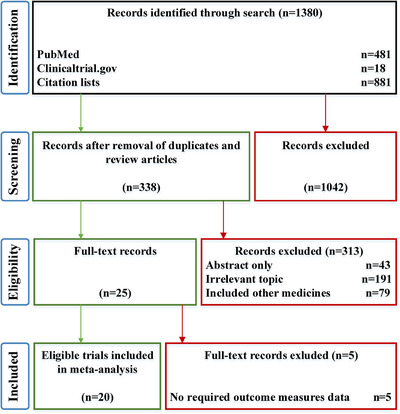
The process of the study identification. Twenty studies were included to evaluate the clinical benefits of neoadjuvant PD‐1/PD‐L1 inhibitors utilization in several tumors

The initial search yielded 1380 articles from PubMed (481), Clinicaltrial.gov (18), and article reference lists (881). After deleting duplication and browsing the abstracts, 338 articles remained. Further, 313 articles were excluded when assessing the eligibility according to the inclusion criteria. Eventually, 20 eligible articles were included in this meta‐analysis.^20^, ^21^, ^22^, ^23^, ^24^, ^25^, ^26^, ^27^, ^28^, ^29^, ^30^, ^31^, ^32^, ^33^, ^34^, ^35^, ^36^, ^37^, ^38^, ^39^ The procedure is demonstrated in Figure 1. Of the 20 articles, seven studies were randomized controlled trials, and the rest studies were single‐arm trials. Due to most of included articles described single‐arm trials, this meta‐analysis had to be a single‐arm analysis. The baseline characteristics of the eligible articles are shown in Table 1. Among these articles, three studies applied PD‐1/PD‐L1 inhibitors plus chemotherapy, five studies applied PD‐1/PD‐L1 inhibitors plus CTLA‐4 inhibitors, and the others used PD‐1/PD‐L1 inhibitors monotherapy. Additionally, nine kinds of tumors were included, and the reported PD‐1‐positive rate varied according to the tumor types and stages. Therefore, the efficacy assessment was analyzed by tumor types to avoid introducing heterogeneity.

**TABLE 1:** The characteristics of included studies

Included studies	Study design	Cancer type	Age	Registration number	Regimen of neoadjuvant therapy	Sample size	PD‐L1 positive
Schmid, P., et al. 2020	Single arm	Triple‐Negative Breast Cancer	48.5 (Mean)	NCT02622074	Pembrolizumab plus Chemotherapy	60	78%
Mittendorf, E. A., et al. 2020	RCT	Triple‐Negative Breast Cancer	51 (Median)	NCT03197935	Atezolizumab plus Chemotherapy versus placebo plus Chemotherapy	164	47%
Loibl, S., et al. 2019	RCT	Triple‐Negative Breast Cancer	50 (Median)	NCT02685059	Atezolizumab plus Chemotherapy versus Placebo plus Chemotherapy	88	69%
Levine, L. S., et al. 2020	Single arm	Melanoma	53 (Mean)	Not reported	Nivolumab	10	Not reported
Huang, A. C., et al. 2019	Single arm	Melanoma	62 (Median)	NCT02434354	Pembrolizumab	29	Not reported
Blank, C. U., et al. 2018	RCT	Melanoma	54 (Median)	NCT02714218	Nivolumab plus ipilimumab	10	40%
Amaria, R. N., et al. 2018	RCT	Melanoma	55 (Median)	NCT02519322	Nivolumab versus Ipilimumab plus Nivolumab	23	67%
Shu, C. A., et al. 2020	Single arm	Lung Cancer	67 (Median)	NCT02716038	Atezolizumab	30	55%
Gao, S., et al. 2020	Single arm	Lung Cancer	62 (Median)	ChiCTR‐OIC‐17013726	Sintilimab	40	55%
Forde, P. M., et al. 2018	Single arm	Lung Cancer	67 (Median)	NCT02259621	Nivolumab	21	Not reported
Uppaluri, R., et al. 2020	Single arm	Head and Neck Cancer	60 (Median)	NCT02296684	Pembrolizumab	36	Not reported
Schoenfeld, J. D., et al. 2020	RCT	Head and Neck Cancer	65.2 (Median)	NCT02919683	Nivolumab versus Nivolumab plus Ipilimumab	29	80%
Ferrarotto, R., et al. 2020	RCT	Head and Neck Cancer	Not reported	NCT03144778	Duralumab	15	93%
Gao, J., et al. 2020	Single arm	Urothelial Carcinoma	71 (Median)	NCT02812420	Durvalumab plus Tremelimumab	28	Not reported
Powles, T., et al. 2019	Single arm	Urothelial Carcinoma	73 (Median)	NCT03800134	Atezolizumab	95	41%
Schalper, K. A., et al. 2019	Single arm	Glioblastoma	54 (Median)	NCT02550249	Nivolumab	29	Not reported
Cloughesy, T. F., et al. 2019	RCT	Glioblastoma	55.4 (Mean)	Not reported	Pembrolizumab	16	Not reported
Necchi, A., et al. 2020	Single arm	Urologic Cancer	66 (Median)	NCT02736266	Pembrolizumab	114	59%
Chalabi, M., et al. 2020	Single arm	Colon Cancer	Not reported	NCT03026140	Nivolumab plus Ipilimumab	40	Not reported
Topalian, S. L., et al. 2020	Single arm	Merkel Cell Carcinoma	68 (Median)	NCT02488759	Nivolumab	39	25.9%

Abbreviation: RCT, randomized controlled trial.

### 3.2 Quality assessment

**FIGURE 2:**
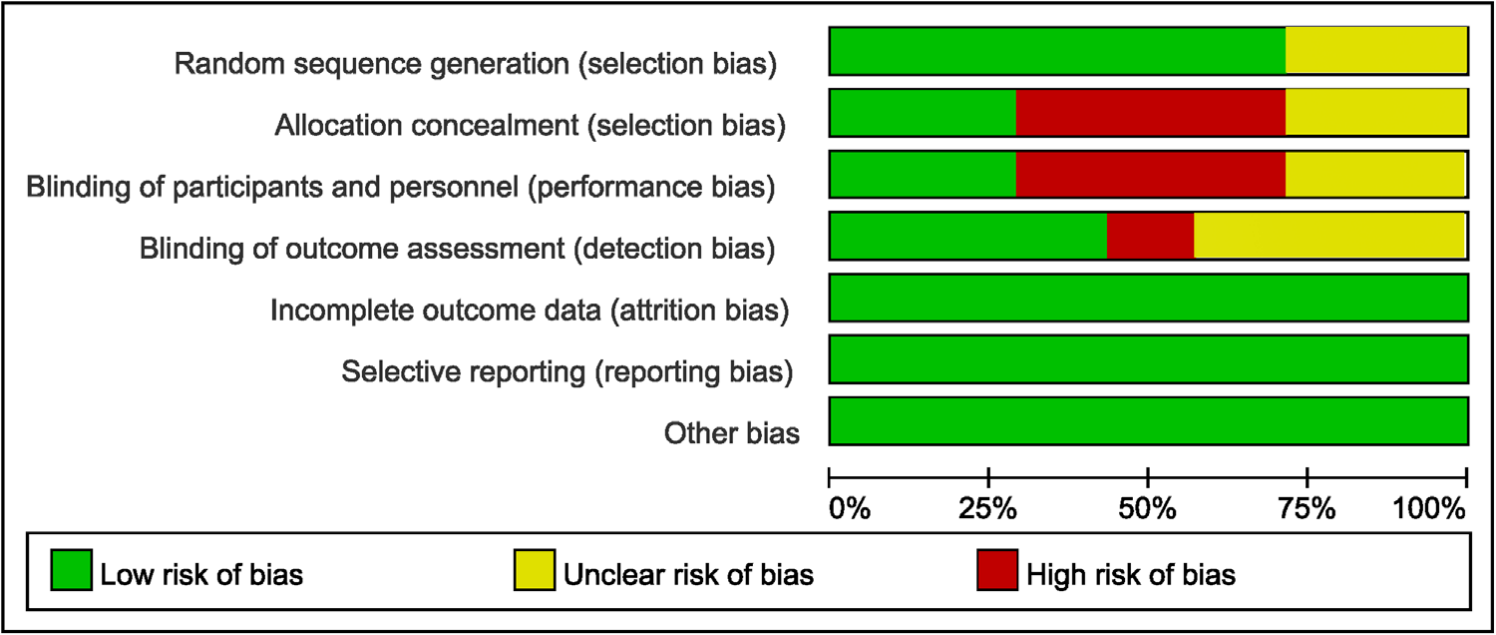
The risk of bias. The allocation concealment and blinding of participants and personnel were not evaluated as low risk. The overall risk of bias was evaluated as low risk

The quality of single‐arm trials was assessed by the MINORS index; the scores are shown in Table S1. All the assessed noncomparative articles were of high quality with scores over 9. The randomized controlled trials were evaluated by the Cochrane Collaboration's risk of bias tool. The result is shown in Figure 2. Apart from selection bias and performance bias, the rest items were evaluated as low risk of bias.

### 3.3 Efficacy

**FIGURE 3:**
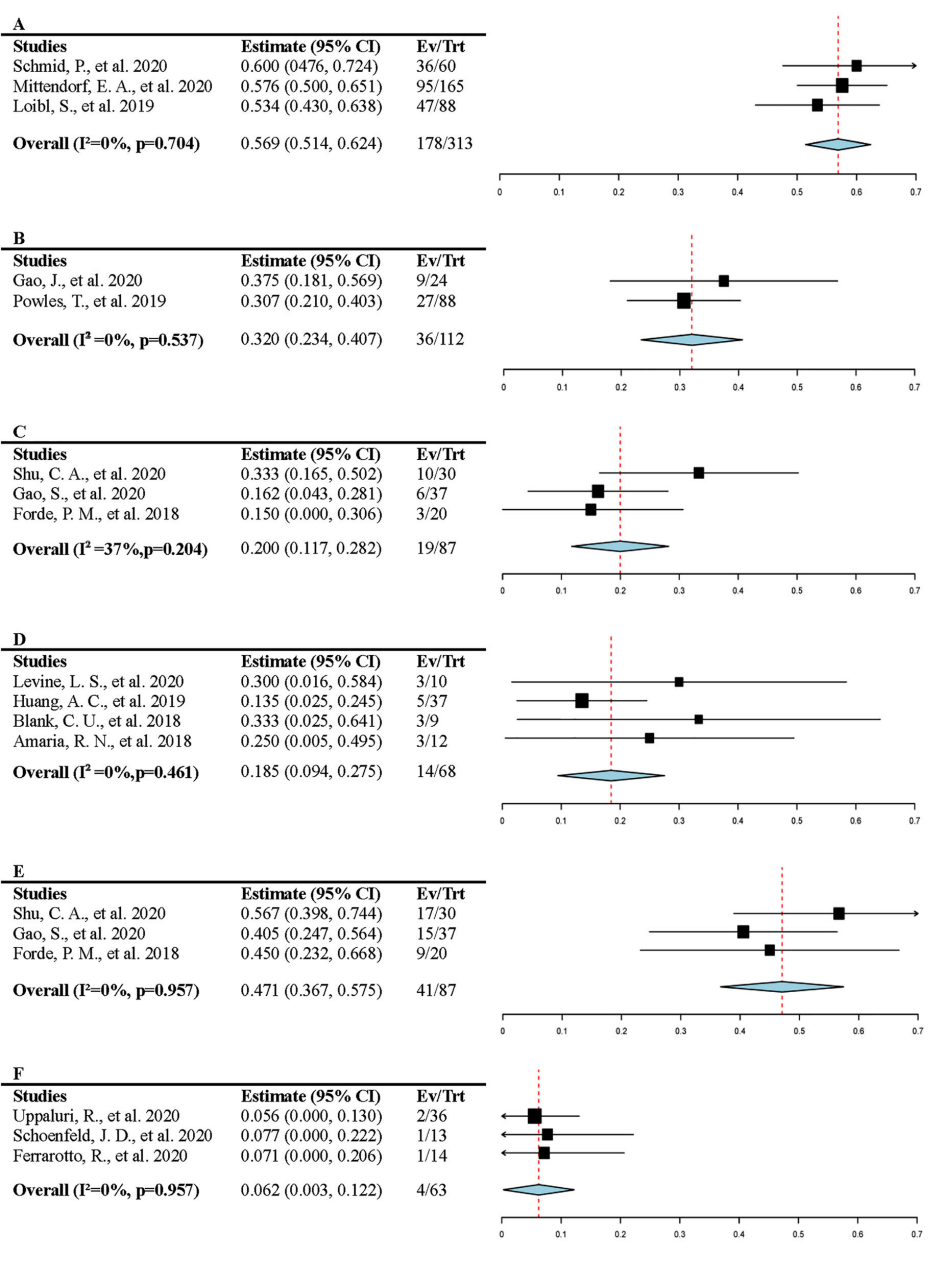
The efficacy of neoadjuvant anti‐PD‐1/PD‐L1 therapy. (A) The complete pathologic response rate in triple‐negative breast cancer patients; the vertical line indicates the overall mean rate (0.569). (B) The complete pathologic response rate in urothelial carcinoma patients; the vertical line indicates the overall mean rate (0.320). (C) The complete pathologic response rate in lung cancer patients; the vertical line indicates the overall mean rate (0.200). (D) The complete pathologic response rate in melanoma patients; the vertical line indicates the overall mean rate (0.185). (E) The major pathological response rate in lung cancer patients; the vertical line indicates the overall mean rate (0.471). (F) The major pathological response rate in head and neck cancer patients; the vertical line indicates the overall mean rate (0.062)

The efficacy of neoadjuvant anti‐PD‐1/PD‐L1 therapy is shown in Figure 3. Three articles reported the pathological CRR of triple‐negative breast cancer after the intervention of neoadjuvant PD‐1/PD‐L1 inhibitors plus chemotherapy, including 313 patients.^37^, ^38^, ^39^ The pooled CRR was 0.569 (95% CI 0.514, 0.624, *I* ^2^ = 0%). Four articles including 68 patients were available of CRR of melanoma after neoadjuvant therapy, two of which combined PD‐1/PD‐L1 inhibitors and CTLA‐4 inhibitors in the neoadjuvant therapy, and the rest used PD‐1/PD‐L1 inhibitors monotherapy. The pooled data were 0.185 (95% CI 0.094, 0.275, *I* ^2^ = 0%).^20^, ^31^, ^35^, ^36^ The response to the neoadjuvant PD‐1/PD‐L1 inhibitors of urothelial carcinoma was assessed in two articles.^29^, ^30^ The pooled CRR was 0.320 (95% CI 0.234, 0.407, *I* ^2^ = 0%). The response of lung cancer was evaluated by the completed response and major response, which were given in three articles.^32^, ^33^, ^34^ The CRR of neoadjuvant anti‐PD‐1/PD‐L1 therapy in lung cancer was 0.200 (95% CI 0.117, 0.282, *I* ^2^ = 37%), and the MRR was 0.471 (95% CI 0.367, 0.575, *I* ^2^ = 0%). The pooled MRR of head and neck cancer was 0.062 (95% CI 0.003, 0.122).^26^, ^27^, ^28^ Overall, PD‐1/PD‐L1 inhibitors provided an obviously reduced risk in the included cancer, and the *I* ^2^ indicated there was low heterogeneity in these studies (Table 2).

**TABLE 2:** The efficacy of neoadjuvant PD‐1/PD‐L1 inhibitor therapy in some tumors

Outcome	Cancer	No. Of studies	No. of participants	Statistical model	Event rate	Lower limit	Upper limit	*I* ^2^
Pathological complete response	Triple‐Negative Breast Cancer	3	313	Fixed model	0.569	0.514	0.624	0%
	Melanoma	4	68	Fixed model	0.185	0.094	0.275	0%
	Lung Cancer	3	87	Fixed model	0.200	0.117	0.282	37%
	Urothelial Carcinoma	2	112	Fixed model	0.320	0.234	0.407	0%
Major pathological response	Lung Cancer	3	87	Fixed model	0.471	0.367	0.575	0%
	Head and Neck Cancer	3	63	Fixed model	0.062	0.003	0.122	0%

### 3.4 Safety

The safety of the neoadjuvant PD‐1/PD‐L1 inhibitors was evaluated by the risk of AEs, which were reported in all of the included articles. Fatigue was the most common adverse event, with 16 articles reporting it. Overall, 253 of 828 patients suffered from fatigue (0.272 95% CI 0.171, 0.402, *I* ^2^ = 87%). Moreover, rashes were reported in 17 studies, occurring in 150 patients (0.180 95% CI 0.131, 0.242, *I* ^2^ = 68%). The risk of nausea was 0.157 (95% CI 0.087, 0.268, *I* ^2^ = 88%), which was a certain risk in this analysis. Other frequent events were alanine aminotransferase increase (0.143 95% CI 0.084, 0.232, *I* ^2^ = 82%), anemia (0.135 95% CI 0.066, 0.256, *I* ^2^ = 88%), diarrhea (0.127 95% CI 0.076, 0.204, *I* ^2^ = 80%), and aspartate aminotransferase increase (0.102 95% CI 0.056, 0.181, *I* ^2^ = 82%). High‐grade adverse events were reported in 18 articles. The most frequent high‐grade adverse events were febrile neutropenia (0.084 95% CI 0.063, 0.112, *I* ^2^ = 85%) and anemia (0.072 95% CI 0.053, 0.098, *I* ^2^ = 88%). The result showed the neoadjuvant anti‐PD‐1/PD‐L1 therapy had tolerable toxicity. However, high heterogeneity existed in almost every result of AEs with certain risks listed above (more than 50%). The pooled AEs and high‐grade AEs are shown in Table S2 and Table S3.

## 4 DISCUSSION

Frequently, the clinical value and quality of current studies on neoadjuvant anti‐PD‐1/PD‐L1 therapy were limited by the small sample size. Therefore, this meta‐analysis was performed to pool the data to evaluate efficacy and safety of the neoadjuvant PD‐1/PD‐L1 inhibitor therapy among several cancers. The result showed that this therapy demonstrated a satisfying response rate and tolerable toxicity among cancers.

PD‐1, an inhibitory receptor from the CD28 family, was widely expressed on several immune cells and nonimmune cells; for instance, activated T cells and vascular endothelial cells.^15^ Based on current understanding, PD‐1 is mainly activated by two ligands, PD‐L1 and programmed cell death ligand 2 (PD‐L2). It is noteworthy that PD‐L1 is constitutively upregulated in various tumors including melanoma, non‐small‐cell lung cancer, head and neck squamous cell carcinoma.^48^, ^49^ Because PD‐1 and PD‐L1 could eliminate cancer cells by immune system suppression, immune checkpoint blockade may be a promising therapy in clinical practice.^50^, ^51^, ^52^ Recent studies have reported the superior efficacy of PD‐1/PD‐L1 inhibitors in lung cancer, melanoma, and other cancers.^53^, ^54^ This meta‐analysis calculated CRR and MRR among cancer patients to evaluate the effectiveness of this therapy. Despite the cancer status and the therapy regimen, the pooled CRR were 0.569 (95% CI 0.514, 0.624), 0.185 (95% CI 0.094, 0.275), 0.320 (95% CI 0.234, 0.407), 0.200 (95% CI 0.117, 0.282), respectively, in triple‐negative breast cancer, melanoma, urothelial carcinoma, and lung cancer. The pooled MRR were 0.471 (95% CI 0.367, 0.575) and 0.062 (95% CI 0.003, 0.122) in lung cancer and head and neck cancer.

This therapy showed the best outcome in triple‐negative breast cancer in this analysis. The possible reason was that the therapy regimen of the included studies was PD‐1 inhibitors plus chemotherapy. Two randomized controlled trials included in this analysis received the superior efficacy of PD‐1 inhibitors plus chemotherapy to pure chemotherapy, and this meta‐analysis confirmed this conclusion (OR: 1.748, 95% CI 1.234, 2.474, *p *= .002, *I* ^2^ = 0%).^37^, ^39^ Melanoma got a CRR of 0.185 (95% CI 0.094, 0.275, *I* ^2^ = 0%). One study compared the efficacy between PD‐L1 plus CTLA‐4 inhibitors and PD‐L1 inhibitors monotherapy. CLTA‐4 repressed the *immune* effect by outcompeting CD28 for its ligand CD80 and CD86, which could be suppressed by CTLA‐4 inhibitors. Current studies have shown that PD‐L1 could exert effects on the CTLA‐4 axis. PD‐L1 can heterodimerize with CD80, thus repressing the interactions of PD‐L1–PD‐1 and CD80–CTLA‐4, but fail to inhibit the combination of CD80 and CD28. Clinical trials concerning several cancers have demonstrated the efficacy of PD‐L1 inhibitors plus CTLA‐4 inhibitors therapy, which could be explained by the above mechanism.^55^, ^56^ However, the existing evidence did not suggest that the neoadjuvant PD‐L1 plus CTLA‐4 inhibitors therapy shows better clinical outcome than PD‐L1 treatment in melanoma, oropharynx cancer, and oral cavity squamous cell carcinoma. Therefore, more studies are required in this area.^27^, ^28^, ^31^

The safety of this therapy also plays a role in patient counseling. Every eligible trial reported a tolerable safety profile, and the most frequent AEs were fatigue, rash, nausea, increasing alanine aminotransferase, anemia, diarrhea, and increasing aspartate aminotransferase, with incidences of 0.272, 0.180, 0.157, 0.143, 0.135, 0.127, and 0.102, respectively. High‐grade AEs were rare, the most common high‐grade AEs were neutropenia and anemia, with incidences of 0.084 and 0.072, respectively. Overall, the most common adverse events occurred in the skin, liver, and blood system. These data and information can be shared with patients before they receive neoadjuvant PD‐1/PD‐L1 inhibitor treatment. Noticing the potential toxicities, doctors and patients can carry out early intervention more directly.

Although this is the first study assessing the efficacy and the safety of neoadjuvant anti‐PD‐1/PD‐L1 therapy systematically, this study has some limitations. First, most of the eligible trials were single‐arm trials, so the superiority over the current therapy could not be evaluated. Second, this study focused on the pathologic response of this therapy, for the postoperative data like survival situation, recurrence risk, R0 resection rate are insufficient in these included studies. Third, with high heterogeneity in adverse events, the meta‐analysis failed to perform subgroup analysis due to the lack of data. Despite of these limitations, this meta‐analysis provided objective clinical information of neoadjuvant anti‐PD‐1/PD‐L1 therapy.

In conclusion, this study revealed an appreciable response rate and tolerable toxicity of neoadjuvant anti‐PD‐1/PD‐L1 therapy in triple‐negative breast cancer, lung cancer, and melanoma. However, more randomized controlled trials are needed to assess the superiority over the current therapy.

## CONFLICT OF INTEREST

The authors declare that there is no conflict of interest.

## ETHIC STATEMENT

Not applicable.

## AUTHOR CONTRIBUTIONS

Zhiyang Li, Lei Deng, Fushen Sha, and Yunuo Zhao collected and analyzed the data and wrote the article. Xuelei Ma and Xin Wu provided the idea. Ting Zhang, Yinan Xiao, and Yating Wang modified the article. Hui Li edited the pictures. Yanjie Zhao performed reference collection. All the authors read and approved the final manuscript.

## Supporting information



Supporting Information

Supporting Information

Supporting Information

## Data Availability

The data included in this study are available upon request from the corresponding author.
