# Supplementary material for: Clinical benefit of neoadjuvant anti‐PD‐1/PD‐L1 utilization among different tumors
Source: MedComm (2020). 2021 Mar 11;2(1):60–8. doi: 10.1002/mco2.61 (PMC8491227; doi:10.1002/mco2.61)
Supplement: Supplementary file 1 — Supporting Information [file MCO2-2-60-s002.pdf]

Appendix A in the Supplement

	Schmid, P., et al. 2020	Adams, S., et al. 2019	Adams, S., et al. 2019	Levine, L. S., et al. 2020	Huang, A. C., et al. 2019	Shu, C. A., et al. 2020	Shu, C. A., et al. 2020	Gao, S., et al. 2020	Forde, P. M., et al. 2018	Uppaluri, R., et al. 2020	Gao, J., et al. 2020	Powles, T., et al. 2019	Schalper, K. A., et al. 2019	Necchi, A., et al. 2020	Chalabi, M., et al. 2020	Topalian, S. L., et al. 2020
A clearly stated aim	2	2	2	2	2	2	2	2	2	2	2	2	2	2	2	2
Inclusion of consecutive patients	2	2	2	2	2	2	2	2	2	2	2	2	2	2	2	2
Prospective collection of data	2	2	2	2	2	2	2	2	2	2	2	2	2	2	2	2
Endpoints appropriate to the aim of the study	2	2	2	2	2	2	2	2	2	2	2	2	2	2	2	2
Unbiased assessment of the study endpoint	0	0	0	0	0	0	0	0	0	0	0	0	0	0	0	0
Follow-up period appropriate to the aim of the study	2	2	2	2	2	2	2	2	2	2	2	2	2	2	2	2
Loss to follow up less than 5%	1	2	2	1	1	2	2	2	2	2	2	2	2	2	2	2
Prospective calculation of the study size	0	0	0	0	0	0	0	0	0	0	0	0	0	0	0	0
